# Conjunctivitis in Children

**Published:** 1908-06-27

**Authors:** J. Herbert Parsons

**Affiliations:** Assistant Surgeon, Royal London Ophthalmic Hospital; Assistant Ophthalmic Surgeon, University College Hospital; Ophthalmic Surgeon, The Hospital for Sick Children, Great Ormond Street.


### Hospital Clinics.

### CONJUNCTIVITIS IN CHILDREN.

By J. HERBERT PARSONS, D.Sc., F.R.C.S., Assistant Surgeon, Royal London Ophthalmic Hospital; Assistant Ophthalmic Surgeon, University College Hospital; Ophthalmic Surgeon, The Hospital for Sick Children, Great Ormond Street.

(Abstract of a Lecture delivered at Great Ormond Street, June 11, 1908.)

I have chosen the subject of conjunctivitis in children for this lecture because it is so extremely important to all of us, and especially so to general practitioners. For instance, one form of it, ophthalmia neonatorum, is an absolutely preventable disease: there is no doubt about that; and yet we find that 10 per cent. or more of all the blind in England are blind because of this disease. The proper prophylaxis of this kind of severe conjunctivitis is therefore a matter of the utmost moment. Yet the individuals are far more numerous in whom defective vision is due to neglect of treatment for other conditions, particularly phlyctenular conjunctivitis.

One may roughly divide the children who attend the eye department of this hospital into four large groups. Those groups are errors of refraction, squints, conjunctivitis, and, lastly, all the other eye diseases lumped together. Even then the last group probably does not exceed in numbers any one of the first three: so it is evident how important the question of conjunctivitis is.

### Ophthalmia Neonatorum.

The first variety of conjunctivitis which is to be considered is ophthalmia neonatorum. This nearly always begins on the third day after birth, showing that in the majority of cases it is a post-natal affection. In a few cases it has been known to start on the same day as the birth, and it has been supposed that it may arise *in utero*; this, however, is a disputed question.

Probably 60 to 70 per cent. of the cases are caused by the gonococcus. Of the rest many are mixed infections, the organisms most frequently present being pneumococci, staphylococci, bacillus coli, and occasionally streptococci. The latter are rare, but are much more dangerous and difficult to treat than the others, even than the gonococcus. As regards severity, after the streptococcal cases, in which the cornea may become entirely necrotic within twelve hours, it is not possible to distinguish clinically between those of gonococcal and those of mixed causation. From the point of view of prophylaxis it is most important to recognise that these non-gonorrhœal cases are numerous, because unfortunately it has occasionally been thought that the adoption of careful and thorough prophylaxis is a stigma on the parents of the child.

In the prophylaxis of this form of ophthalmia there is no doubt that the old Crédé method, modified by reduction of the strength of the silver nitrate solution to one per cent., is of very definite value as a preventive measure. All other drugs, such as argyrol, protargol, and so on, are less efficacious than ordinary nitrate of silver. For the reasons I have already given it is essential to take this prophylactic measure in every case.

In the treatment of existing ophthalmia the first requisite is to discover the condition of the cornea, which may be very seriously damaged before one sees the patient at all. It is not easy to examine the cornea without using a certain amount of force, so that extreme care must always be used: otherwise a cornea which is so affected as to have nearly given way may be ruptured by the manipulations. In children particularly it is often most difficult to get the lids satisfactorily open without exerting some pressure on the globe. With the fingers it is necessary to put the thumbs quite close to the margins of the lids and maintain slight pressure through them on the globe while the lids are separated: otherwise the lids are merely everted, and their swollen conjunctival surfaces meet across the eyeball and prevent a view of the cornea. It is, therefore, advisable on the first examination to use retractors, by which the lids can be lifted away from the globe as they are separated. After the first examination one knows the conditions present, and may on that account have less dread of the bad result which may follow slight pressure on the globe.

As to diagnosis, the other forms of conjunctivitis which can be met with at an early age are best distinguished from ophthalmia neonatorum by the history. But it must not be forgotten that the quantity of discharge at the onset of the disease may at times be very slight, so that it may be some time after birth before severe symptoms develop. Still, the discharge from other forms of conjunctivitis is very seldom pure pus as in ophthalmia neonatorum, but much more often thick mucus or muco-pus. Indeed, the best term for the group of cases which was formerly termed catarrhal is muco-purulent conjunctivitis. The difference between the two sorts of discharge is very readily appreciated when it is seen.

### Treatment of Purulent Ophthalmia.

For ophthalmia neonatorum, as in its prevention, the one remedy is silver nitrate. First of all comes the thorough cleansing of the conjunctival sac not less than every two hours with weak perchloride of mercury lotion, or boracic acid, or sterile saline solution. This is most important, and the volume of water used in the washing away of all discharge is the principal consideration. The next point is the use of a silver salt, and here, as in prophylaxis, I prefer the actual nitrate to the organic compounds. I use a two-per-cent. solution painted on to the everted lids once a day. The effect of this is to form on the surface of the conjunctiva a thin film of slough, which is presently thrown off in the discharge and washed away. In this slough the organisms are entangled, though not killed, and those which are contained in the epithelial cells are removed at the same time.

The reason why the cornea suffers so severely in gonorrhœal conjunctivitis is that the gonococcus can occupy the normal undamaged epithelial cell, whereas other organisms cannot do so. Thus in the case of the pneumococcus, often present in the corneal ulcers of adults, there must be some abrasion before the entry of the organism is possible. One sees, therefore, that silver nitrate, by causing the destruction of the superficial layer of epithelial cells, backs up the results of frequent douching as an effective method of getting rid of the micro-organisms which have invaded the conjunctiva.

In those cases in which the cornea is already ulcerated, the instillation of atropine is required just as in any other case of corneal ulceration.

I do not think that any other strength of silver is so satisfactory: for weaker solutions do not cause the formation of a slough and are merely astringent; while stronger ones cause deeper sloughing than one wants, are deleterious to the cornea also, and lower its vitality in a serious manner. *Once* a day is quite sufficient, no matter how bad the case may be. The process of creating a slough, of throwing it off, and of gradual restoration of the epithelium takes a considerable time; and if the silver is used more frequently than once a day too much reaction and hyperæmia are caused. Thereby the amount of discharge is also increased consequent on the increased vascularity produced.

### Phlyctenular Conjunctivitis.

One of the commonest forms of conjunctivitis with which we have to deal in children is the phlyctenular variety. This is very commonly associated with mucopurulent conjunctivitis, but the distinction between the two is often overlooked. The phlyctenule is usually in the neighbourhood of the limbus, or the corneo-scleral junction. Less often it is at some little distance from the corneal margin; in this case there will be seen a leash of blood-vessels running up to it, and there may be more than one phlyctenule. If there is no other affection of the conjunctiva, there will then be little or no blepharospasm, and but slight irritation; but when the limbus is affected there is considerably more discomfort as a rule, though with little reflex blepharospasm. When the phlyctenule is near the corneal margin, and there is much discharge and irritation, the result is usually due to the rubbing in by the child of staphylococci from the skin with his own fingers, so that he gets a mucopurulent conjunctivitis as well as the phlyctenular lesion.

An ordinary phlyctenule may lose its surface epithelium, and give rise to a small ulcer of the cornea or conjunctiva. But when there is the complication of mucopurulent conjunctivitis the chances of ulceration and of infection of the ulcers are very much increased, and it is in these cases that the extreme forms of blepharospasm are seen. This reflex is not a light reflex, but is due directly to the inflammation and consequent irritation of the endings of the fifth nerve.

The causation of phlyctenular conjunctivitis is important, both theoretically and from the point of view of treatment. Bacteriologists came to the conclusion that it is a staphylococcal affair, because those organisms are most commonly found in the conjunctivæ of patients who suffer from this disease; but these staphylococci are in reality the result of secondary infection, and in point of fact it is not known what the exact cause is. It was long thought that the disease especially affects children of the tuberculous type; but there is no doubt that it occurs in many children who have no definite clinical signs of tuberculosis of any kind. Still, it is true that the nearest approach to phlyctens—not a very close one—is produced by injecting dead tubercle bacilli into the circulation of rabbits. Some of Wright's observations also support the idea that phlyctenular conjunctivitis is a toxic effect of the products of tubercle bacilli. The one thing that is fairly certain is that phlyctens are not caused by staphylococci.

Eczema of the skin is very common in the subjects of phlyctenular conjunctivitis. This may affect the face, and there it may be partly due to the irritation of tears and rubbing; but in many cases the skin affected is not that of the face at all. In Germany the disease is even known as eczematous conjunctivitis, so frequent is the association of the two conditions. Eczema, when it exists, assists in the development of any staphylococcic infection that may be derived from the skin.

The chief danger of phlyctenular conjunctivitis is, as with ophthalmia neonatorum, though in a totally different manner, the destruction of the cornea. For there is great liability for the process to affect the cornea in just the same way as it does the conjunctiva: the consequence of this is a phlyctenular keratitis, which is very superficial to start with, and whose evil effects are chiefly due to the secondary infections so easily implanted on an abraded cornea.

There is one type of phlycten which may lead to damage without any secondary infection—namely, the fascicular ulcer, which travels across the cornea, healing as it goes on the side nearest the limbus. In its progress it drags its leash of blood-vessels behind it, and so causes opacity. When it finally stops, this opacity is densest at the spot where ulceration last existed; hence, if seen for the first time when the ulcer has already reached the centre of the cornea, it may be advisable to allow it to proceed a little further before commencing energetic efforts to cure it.

As regards treatment, it is of the utmost importance not to neglect general treatment. In specialties connected with local regions one is often apt to remember only local measures, but this is a tendency which in eye diseases it is especially necessary to avoid. Particularly, fresh air must be secured: it is undoubtedly a fact that all cases of conjunctivitis, whether in children or adults, do best out of doors, perhaps because of freer lachrymation and sweeping away of discharge. If there is much blepharospasm, both eyes must be shaded from the light.

When there is no corneal complication there is no indication whatever for atropine. One of the most difficult problems in ophthalmic medicine is when to order and when not to order this drug; but in simple phlyctenular conjunctivitis it certainly should not be used. If there are any phlyctens involving the cornea directly, then atropine is required on the ordinary principles followed in treating corneal ulcer.

The cases do best when some slightly stimulating application is used, and some form of the yellow oxide of mercury in an ointment, though a well-worn prescription, is still one of the best remedies we have. It acts probably by causing a certain amount of hyperæmia of the conjunctiva, so stimulating the lymph flow in the deeper tissues. There is an old-fashioned form of treatment not often used now, but very valuable in certain cases, and that is finely divided calomel applied with a camel-hair brush. It was once thought that the effects of this might be purely mechanical, and powdered glass in as finely divided a condition was tried instead; but the latter was not successful, so it seems as if the calomel really has some specific beneficial action. In the general treatment of these children one sometimes orders tonics containing iodide of iron; it must be remembered that if any kind of iodide is being taken calomel must under no circumstances be applied to the conjunctiva.

As regards lotions, the washing away of discharge and keeping the conjunctiva clean are the important matters, so that there is not much to choose between the ordinary lotions. But when there is secondary staphylococcic infection and mucopurulent conjunctivitis, weak mercurial lotions, such as one in eight thousand perchloride of mercury, seem to me to act better than boracic or other solutions.

### Diphtheritic Conjunctivitis.

There are other varieties of conjunctivitis, but I can hardly do more than mention to you diphtheritic conjunctivitis now. The point about this upon which I wish to lay stress is that every membranous conjunctivitis is not of diphtherial origin. It has been quite definitely shown that in the severest forms of membranous conjunctivitis there may be no diphtheria bacilli at all. In these cases there are often found pneumococci, gonococci, or other organisms. Whenever there is any doubt the proper course is to give antitoxin.

It is quite unusual to find that the patients themselves have diphtheria of the faucial regions, in my experience at least; though the infection is doubtless derived originally from an ordinary case. It is, therefore, essential to have a bacterial examination made of the conjunctival exudate; as in faucial diphtheria, there is a bacillus found which is morphologically indistinguishable from the bacillus diphtheriæ, and cultural methods are necessary to establish the diagnosis. I personally order the use of anti-toxin locally, as well as by subcutaneous injection in these patients, and I have found such local treatment beneficial for this form of conjunctivitis.

